# Biochemical and Metabolomic Changes after Electromagnetic Hyperthermia Exposure to Treat Colorectal Cancer Liver Implants in Rats

**DOI:** 10.3390/nano11051318

**Published:** 2021-05-17

**Authors:** Borja Herrero de la Parte, Mireia Irazola, Jorge Pérez-Muñoz, Irati Rodrigo, Sira Iturrizaga Correcher, Carmen Mar Medina, Kepa Castro, Nestor Etxebarria, Fernando Plazaola, Jose Ángel García, Ignacio García-Alonso, Jose Javier Echevarría-Uraga

**Affiliations:** 1 Department of Surgery and Radiology and Physical Medicine, University of The Basque Country, ES48940 Leioa, Biscay, Spain; ignacio.galonso@ehu.eus; 2 Biocruces Bizkaia Health Research Institute, ES48903 Barakaldo, Biscay, Spain; jorge.perezmunoz14@gmail.com (J.P.-M.); kepa.castro@ehu.eus (K.C.); nestor.etxebarria@ehu.eus (N.E.); fernando.plazaola@ehu.eus (F.P.); joseangel.garcia@ehu.eus (J.Á.G.); josejavier.echevarriauraga@osakidetza.eus (J.J.E.-U.); 3 Department of Analytical Chemistry, University of the Basque Country, ES48940 Leioa, Biscay, Spain; 4 Research Centre for Experimental Marine Biology & Biotechnology, ES48620 Plentzia, Biscay, Spain; 5 Department of Electricity and Electronics, University of The Basque Country, ES48940 Leioa, Biscay, Spain; iratirodrigo@gmail.com; 6 Department of Clinical Analyses, Osakidetza Basque Health Service, Galdakao-Usansolo Hospital, ES48960 Galdakao, Biscay, Spain; sira.iturrizagacorrecher@osakidetza.eus (S.I.C.); mariadelcarmen.marmedina@osakidetza.eus (C.M.M.); 7 Department of Physics, University of the Basque Country, ES48940 Leioa, Biscay, Spain; 8 Department of Radiology, Osakidetza Basque Health Service, Galdakao-Usansolo Hospital, ES48960 Galdakao, Biscay, Spain

**Keywords:** hyperthermia, liver function, metabolomics, animal model, liver metastases

## Abstract

Background: Hyperthermia (HT) therapy still remains relatively unknown, in terms of both its biological and therapeutic effects. This work aims to analyze the effects of exposure to HT, such as that required in anti-tumor magnetic hyperthermia therapies, using metabolomic and serum parameters routinely analyzed in clinical practice. Methods: WAG/RigHsd rats were assigned to the different experimental groups needed to emulate all of the procedures involved in the treatment of liver metastases by HT. Twelve hours or ten days after the electromagnetic HT (606 kHz and 14 kA/m during 21 min), blood samples were retrieved and liver samples were obtained. 1H-nuclear-magnetic-resonance spectroscopy (1H-NMR) was used to search for possible diagnostic biomarkers of HT effects on the rat liver tissue. All of the data obtained from the hydrophilic fraction of the tissues were analyzed and modeled using chemometric tools. Results: Hepatic enzyme levels were significantly increased in animals that underwent hyperthermia after 12 h, but 10 d later they could not be detected anymore. The metabolomic profile (main metabolic differences were found in phosphatidylcholine, taurine, glucose, lactate and pyruvate, among others) also showed that the therapy significantly altered metabolism in the liver within 12 h (with two different patterns); however, those changes reverted to a control-profile pattern after 10 days. Conclusions: Magnetic hyperthermia could be considered as a safe therapy to treat liver metastases, since it does not induce irreversible physiological changes after application.

## 1. Introduction

The most frequent tumor masses of liver tissue are metastases, in occidental countries even being more frequent than primary hepatic tumors [1,2]. Most of them have gastrointestinal origins; however, they can also be derived from other types of cancer, such as breast carcinoma, lung cancer, genitourinary cancers from kidney or adrenal origin, gynecological cancers from ovarian or uterine origin, melanoma, or sarcomas [1,2,3]. Primary colorectal cancer (CRC) tumors are the main origin of CRC liver metastases (CRCLM). They are also the main site of metastasis relapse [1,3,4].

Nowadays, hepatic resection still remains the standard curative treatment for patients with resectables CRCLM. However, for those patients not suitable for liver resection surgery, other therapies are available, such as radiofrequency ablation (RFA), chemoembolization (CE), or even hyperthermia [5,6], enhanced or not by magnetic nanoparticles (NP).

Hyperthermia is a less common therapeutic option than other therapies. Earlier studies on the application of NP-based antitumor HT, also known as magnetic hyperthermia (MHT), therapies date back to 1957, when Gilchrist et al. sought a new therapeutic approach for the destruction of remaining lymph node metastases after the removal of primary CRC tumors. [7].

HT causes cellular stress, which triggers the activation of various extra- and intracellular processes that lead to protein denaturation, incorrect folding, aggregation or cross-linking of DNA strands [8]. This results in cell death by apoptosis, unlike other thermal therapies such as RFA, which generates temperatures above 46 °C and therefore induces necrosis, carbonization or coagulation of the tissue [9]. At the tissue level, HT causes changes to the pH levels, to the perfusion and oxygenation of the tumor microenvironment, and therefore to the tumor itself [10].

Our research group has solid experience in the field of hyperthermia. For more than 10 years we have been focusing on the development of a magnetic hyperthermia treatment for CRCLM. Along these years, in vitro, ex vivo and in vivo experiments were carried out to study and develop surgical procedures for NP infusion, the synthesis of magnetic NP and their functionalization, the design of electromagnet applicator prototypes and diagnosis techniques. Many research works and conference communications are part of the outcome of this project [11,12,13,14,15,16,17,18,19,20,21,22].

Many studies have been conducted since then, on various experimental models and clinical trials, with different electromagnetic pulses and various types of NP [23,24,25,26,27,28,29,30], assessing the therapeutic outcome of MHT. However, the effects on the correct functioning of the organism are not well known, and several works point out the importance of a better knowledge of these effects [8,31,32,33].

Our aim was to assess whether exposure to EMF, such as that required for MHT therapy, could affect the normal functions of the organism, focusing most closely on liver function.

## 2. Materials and Methods

All of the procedures used in this study were performed in strict accordance with the recommendations of the current national legislation on animal and biological agents’ experimentation. The protocols were approved by the Ethics Committee on Animal Experimentation (CEEA) of the University of the Basque Country (UPV/EHU) (ref. No. CEEA/407/2015 and M20/2016/023).

### 2.1. Tumor Induction and Intra-Arterial Infusion

Seventy-six WAG/RijHsd rats, weighing 250–280 g, were used. The animals were housed in a temperature and humidity-controlled room with 12 h light/dark cycles and free access to water and a standard laboratory diet. To induce liver metastases, CC531 syngeneic cells were injected under US guidance into the left lateral liver lobe of seventy rats. The other six animals were used as a control group.

For tumor development, the protocol followed was the one previously described by our group [13,15]. Briefly, 25,000 colorectal cancer cells (CC531 cell line, Eppelheim, Baden-Württemberg, Germany), were suspended in 0.05 mL of Hank’s solution; under 1.5% isoflurane anesthesia the animals were administered a dose of meloxicam (2 mg/kg, sc) and upper laparotomy was performed to expose the distal end of the left lateral hepatic lobe (LLL) and the cell suspension was subcapsular injected. Then, the animals were let to recover under a heat lamp, and buprenorphine (0.05 mg/kg sc) was administered every 12 h for 3 d. Tumors were allowed to develop for 21 d; then an ultrasound examination was performed to identify tumor-bearing animals. Six tumor-bearing animals were randomly distributed into each of the experimental groups (54 animals in total); the other 22 animals, which did not develop tumors, were then euthanized.

To mimic the infusion of therapeutic compounds, saline was infused 7 d later (that is, 28 d after cell inoculation). Under 1.5% isoflurane anesthesia, those sham-operated animals were placed over a thermal pad at 37 °C, were given a dose of meloxicam (2 mg/kg, sc) and a middle laparotomy was performed to expose the splenic artery. Then, a perfluorocarbon catheter (Harvard Apparatus, Holliston, MA, USA) was carefully inserted through the splenic artery into the hepatic artery, and saline was slowly administered (3 to 5 min) [15]. Once the infusion was accomplished, the abdominal organs were reintroduced into the abdominal cavity, the laparotomy was closed, and, finally, the rats were left under a heat-lamp to recover from anesthesia.

### 2.2. Thermal Therapy

The rats were exposed to focused HT inside an electromagnetic applicator (EA); in sham-operated groups, HT was performed twelve hours after sham surgery. Under diazepam (15 mg/kg; ip), ketamine (80 mg/kg; ip) and medetomidine (0.5 mg/kg; ip) anesthesia, a 2 cm subxiphoid laparotomy was performed in order to expose the left lateral lobe (both the tumoral mass and the healthy liver parenchyma). A thermal probe was placed within the tumor, a second thermal probe was placed between healthy liver lobes, and the last probe was placed in the rectum. Another external thermal probe was placed inside the EA to monitor the device’s temperature. Once the thermal probes were connected to an electronic device to register the thermal oscillations, and the animal was placed inside the EA.

Hyperthermia was induced by applying a magnetic field of 606 kHz and 14 kA/m for 21 min. To avoid overheating, which would result in liver or other organs damage, once the thermal probe placed on healthy liver parenchyma reached 43 °C the intensity was automatically ranged between 4 and 14 kA/m by an on–off controller in order to maintain the liver temperature constant at 43 °C until the treatment was accomplished. Then, the rats were given a single dose of meloxicam (2 mg/kg sc) and the surgical incision was closed as previously described. The animals were placed under an infrared heat lamp bulb and were monitored for signs of pain or distress until they were fully recovered; buprenorphine (0.05 mg/kg sc) was administered every 12 h for 3 d.

### 2.3. Blood and Tissue Collection

Twelve hours or ten days after HT (depending on the experimental group), both liver tissue and blood samples were obtained. Under isoflurane anesthesia, 5–6 mL of blood was collected from the inferior cava vein (ICV) and serum was obtained by centrifugation. Serum samples were frozen in liquid nitrogen and then stored at −84 °C until processed.

Then, two tissue samples of both tumor and healthy livers were retrieved. A sample of each tissue was immersed in 4% paraformaldehyde (for pathology) and the other one was immediately frozen in liquid nitrogen (for NMR-based metabolomic analysis).

### 2.4. Biochemical Analysis

To analyze the potential damage induced by hyperthermia therapy, we studied enzymes that show abnormalities in the liver function (alanine transaminase (ALT), aspartate transaminase (AST), and alkaline phosphatase (ALP)). In addition, other enzyme determinations were also carried out in order to be able to have an idea of the general state of the organism, such as markers of tissue (creatine kinase (CK), and lactate dehydrogenase (LDH), renal (creatinine (Cr) and pancreatic (amylase)) damage.

Serum samples were analyzed in a Cobas^®^ 8000 modular clinical analyzer, equipped with a Cobas c702 module (Hoffmann-La Roche, Basel, Basel-Stadt, Switzerland), and kits for enzymes’ quantification (all from Roche Diagnostics GMBH, Rotkreuz, Zug, Switzerland).

### 2.5. Metabolomic Studies

To determine the metabolomic changes, the methodology described by Professor Mark Viant and co-workers [34,35] was applied. One hundred mg of each sample were obtained and the acquired 1H-NMR spectra were imported to MATLAB using the RBNMR function and processed using the PLS toolbox. Then, the data were pre-processed, using COW alignment, variable selection (water and TMSPA signal as well as spectral areas without peaks were removed), logarithm (log10) transformation, normalization (taking into account all variables area) and mean center and orthogonal signal correction (OSC). Finally, PCA analysis was performed in order to obtain a general idea of how the samples were distributed and to check if any outlier should be removed. If PCA analysis showed a clustering of the samples, then PLSDA and/or OPLS-DA (PLS-DA with OSC) analysis was performed. By means of these supervised chemometric tools, we wanted to enhance the differences among the groups, trying to identify the key variables of the clustering on PLS projections (VIP). In order to ensure the data treatment and analysis procedure (to get meaningful results) a quality control sample batch was used [36]. In all cases, the quality control sample batch showed a perfect clustering (in PCA and (O)PLS-DA analysis) and it was clearly distinguished from the rest of the data. Finally, VIP data were studied to obtain the metabolome profile for each sample group. VIP peaks were exported from MATLAB to EXCEL to obtain the heatmaps and graphical representations of the identified metabolites in order to obtain a fast and clear idea of the most meaningful differences among the sample groups. The identification was performed based on the literature [37,38,39,40,41] and free data bases available on the internet [42,43,44].

### 2.6. Statistical Analyses for Serum Samples

Statistical analyses were performed with Prism^®^ software (GraphPad Software Inc., San Diego, CA, USA).

Biochemical levels were presented as mean ± standard deviation, as the data were normally distributed (analyzed by the Kolmogorov–Smirnov normality test). Comparisons between groups were performed using an analysis of variance (ANOVA) and Dunnett’s multiple comparisons test.

## 3. Results

### 3.1. Biochemical Changes

**Figure 1:**
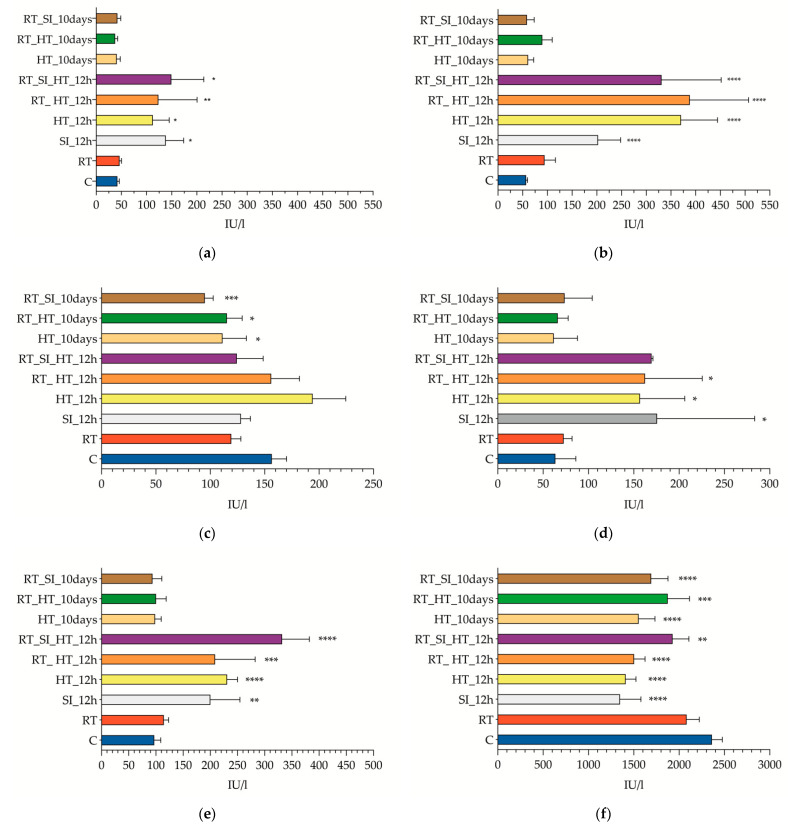
Biochemical serum levels. Mean and standard deviation values of the blood plasma levels of the different enzymes analyzed: alanine transaminase (ALT) (**a**), aspartate transaminase (AST) (**b**), alkaline phosphatase (ALP) (**c**), lactate dehydrogenase (LDH) (**d**), creatine kinase (CK) (**e**) and amylase (**f**). The units of measurement for each enzyme are shown in brackets as international units per liter (IU/L). Key information to understand the abbreviations: control (**c**), rat with CRCLM (RT), hyperthermia therapy procedure (HT), sham surgery (SI) and period of time elapsed since the procedure (12 h or 10 d).

When investigating the changes in the enzymes that mark liver damage, including ALT, AST and ALP (Figure 1A–C, respectively), we noticed that both ALT and AST exhibited similar trends, while ALP showed completely opposite patterns to the previous ones. ALT reached statistically significant peak levels at 12 h post procedure in all experimental groups (*p* < 0.05) when comparing to control animals. These values were almost three times higher than the reference values of the control group (42 ± 4.6 vs. 129.6 ± 52.87 IU/L). AST showed similar patterns in both the 12 h and 10 d samples. In samples obtained 12 h after the last procedure, the animals of the sham group (RT_SI_HT_12h) showed AST levels that were 3.5 times higher than the control ones (202 ± 45.8 vs. 57 ± 3.61 IU/L, *p* < 0.05); whereas when animals were exposed to EMF (HT-12h, RT_HT-12h, or RT_SI_HT_12h group) the AST values were up to six times higher (370 ± 73.9, 388 ± 119, and 331 ± 121 IU/L, respectively; *p* < 0.001). ALP showed an entirely different pattern. Samples collected 10 d after the last procedure had 30–40% lower ALP levels than control ones (66.12 ± 22.2 IU/L vs. 158 ± 16.5 IU/L; *p* < 0.05); whereas when comparing ALP levels in blood samples collected 12 h after the last procedure, we found no significant differences.

When we analyze the evolution of LDH levels (Figure 1D), a similar trend was observed to that of AST and ALT. Samples obtained within 12 h after the last procedure revealed a significant increase (63.7 ± 27.6 vs. 165.2 ± 61.9 IU/L; *p* < 0.05). LDH values returned to normal 10 d later (*p* > 0.05). CK also showed a similar pattern (Figure 1E). Serum samples obtained 12 h after the last procedure CK values were doubled (*p* < 0.01), returning to normal values 10 d later.

Amylase levels in all of the experimental groups showed a significant decrease when comparing to control values (2361 ± 115 IU/L) (Figure 1F). Finally, creatinine showed no variation in serum levels in any of the experimental groups analyzed (Table A1).

### 3.2. Metabolomic Changes in Hepatic Tissue

The first step was to characterize the samples and to establish the basal level of the tissues without any surgery or HT treatment. In this case, hepatic tissue of healthy rats (C) and both hepatic and tumor tissue of tumor-bearing rats (HTRT and TC, respectively) were considered. This task gave us the chance to obtain the metabolome profiles of the different tissues.

**Figure 2:**
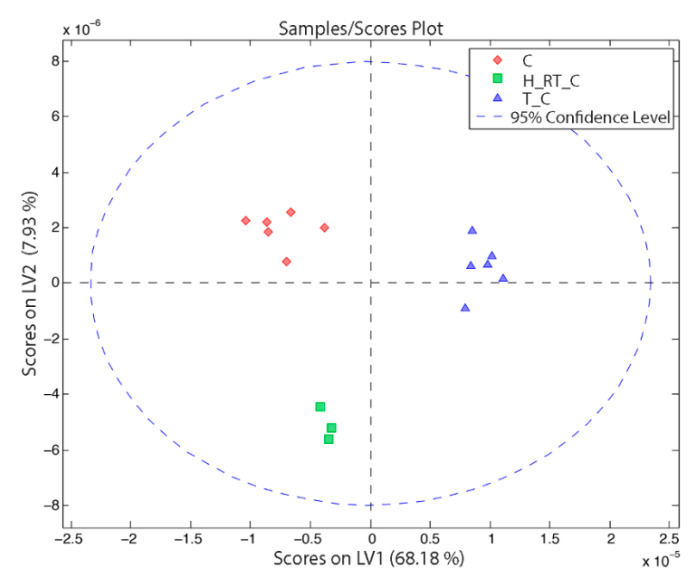
PLS-DA scores from a dataset that combines healthy rats (C) and rats that suffered CRCLM (H_RT_C and T_C). Key information to understand the abbreviations: control (C), hepatic tissue (H), rat with CRCLM (RT) and tumor (T). LV (latent variable) is the same as PC (principal component).

**Figure 3:**
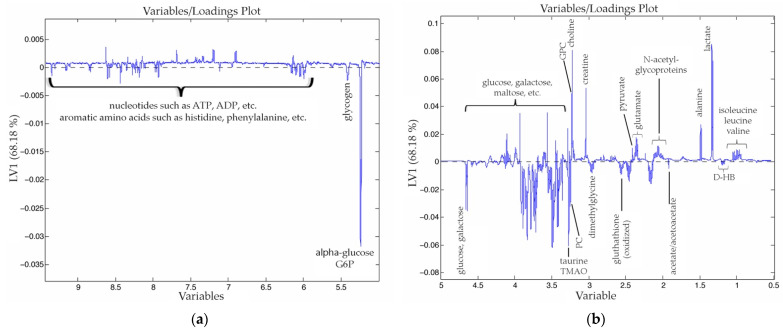
Loadings of the first PC (68.28% of the variance explained) of the PLS-DA analysis obtained from a dataset that combines healthy rats (C) and rats that suffer CRCLM (HRTC and TC). To make it easier to observe the peaks in the graph, it has been divided in two parts. In the first half of the graph (**a**) the *y* axis is displaced downwards to include α-glucose and glucose-6-phosphate (G6P), while in the second half (**b**) it is displaced upwards.

In Figure 2 and Figure 3a,b, we show the scores and the loadings obtained from the PLS-DA analysis of the dataset. The first PC, which explains 68% of the model variance, clearly differentiated the tumor tissue from the healthy hepatic tissue. As we can observe in the Figure 3, tumor tissue was characterized by a higher concentration of lactate, leucine, alanine, glutamate, pyruvate, choline and creatine, among others. Meanwhile, healthy hepatic tissue was characterized by a higher concentration of acetoacetate, D-hydroxybutyrate, dimethylglycine, glucose, galactose and glycogen, among others.

**Figure 4:**
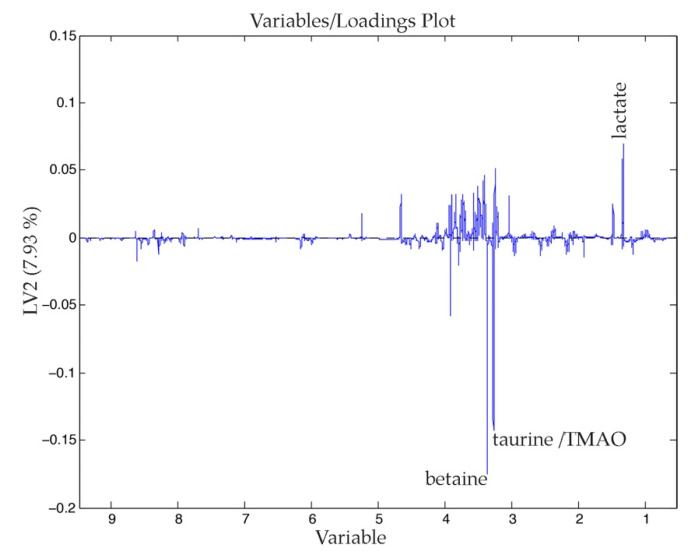
Loadings of the second PC (7.93% of the variance explained) of the PLS-DA analysis obtained from a dataset that combines healthy rats (C) and rats that suffer CRCLM (HRTC and TC).

The second PC, which explains 8% of the model variance, differentiated the hepatic tissue of healthy rats from rats that presented CRCLM. The main differences between these tissues are show in the Figure 4. The metabolites that had a higher weight in this differentiation were lactate, which characterized the healthy rats (with a higher concentration), and betaine and taurine or trimethylamine-N-oxide (TMAO), which characterized tumor-bearing rats. In Table A2 we have collected the normalized VIP values, taking the healthy hepatic tissue samples as a reference. From the relative values, we modeled colors to show the level of the variations. For instance, the signal of the D-hydroxybutyrate increased twice in the case of the hepatic tissue from tumor-bearing rats, whereas the signal in the tumor was much lower.

**Figure 5:**
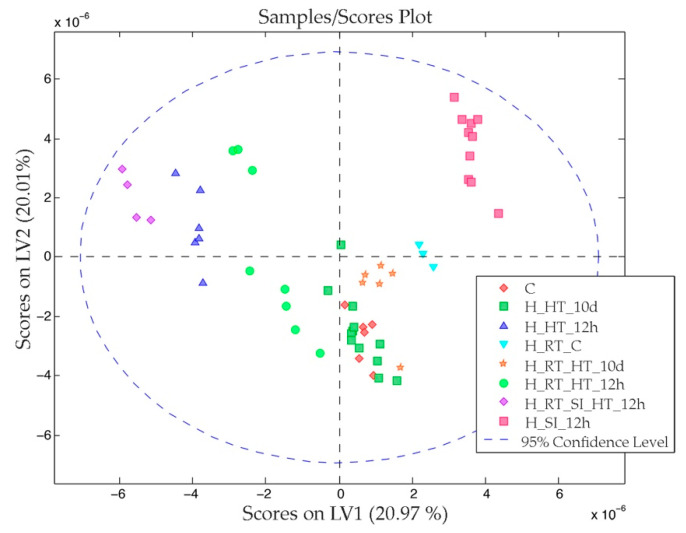
OPLS-DA scores from a dataset that combines healthy rats (C, HHT12h, HSI12h and HHT10d) and rats that suffered CRCLM (HRTC, HRTHT12h, HRTSIHT12h and HRTHT10d) under different procedures. Key information to understand the abbreviations: control (C), hepatic tissue (H), rat with CRCLM (RT), hyperthermia therapy procedure (HT), sham surgery (SI) and period of time elapsed since the procedure (12 h or 10 d).

In Figure 5, we show the score plot of the OPLS-DA model built with the dataset that contains hepatic tissue of healthy rats and tumor-bearing rats that underwent the different procedures. The model was built using three PC, and the three PC explain 54% of the variance of the x matrix (variables) and 34% of the variance of the y matrix (classes). The samples collected 12 h after exposing the animals to HT are located at the left side of the scores plot. By contrast, the samples from animals that were given the sham operation (RT_SI_HT_12h) are located clearly at the upper right-hand side of the scores plot. In addition to this, the samples from animals receiving both treatments are located at the left edge of the data distribution, being the most distant samples from the control samples. Finally, it can be observed that the control samples and the samples from HT group (on the 10th day) are clustered together; this data clustering shows that the rats fully recovered the basal metabolic state after 10 d.

**Figure 6:**
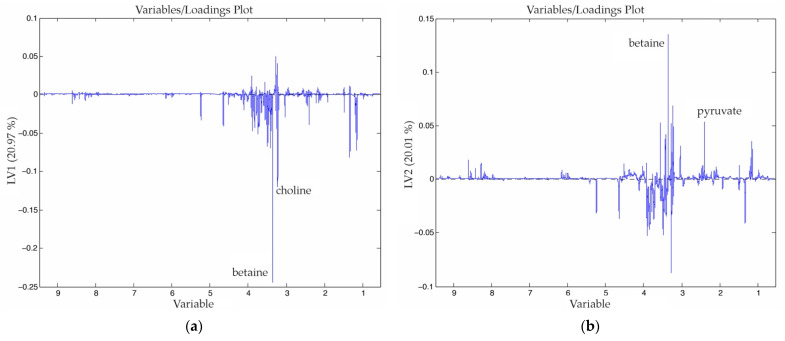
Loadings of the first (**a**) and second (**b**) PC of the OPLS-DA analysis obtained from a dataset that combines healthy rats and rats that suffered CRCLM under different procedures.

Regarding to the loadings of the model (Figure 6), in the first PC, two signals were increased (betaine and choline) that characterize the positive right-hand side of the score plot (Figure 6a). In the second PC (Figure 6b), we find that betaine and pyruvate signals increased.

The most meaningful metabolites identified in the VIPs are summarized in the Table A3, as we proceeded in the previous case. Additionally, to provide further insight into possible interactions, some of the metabolites shown in Table A3 are plotted in Figures S1 and S2. At first glance, metabolites such as glucose, lactate, fumarate, D-hydroxybutyrate, glutathione (oxidized), betaine and L-asparagine show the same pattern (Figures S1 and S2). We observed an important metabolic increment in the treated rats after 12 h, with higher levels of the mentioned metabolites. The lactate/pyruvate ratio (L:P) and the lactate are plotted in Figure S3.

### 3.3. Metabolomic Changes of Tumor Tissue

**Figure 7:**
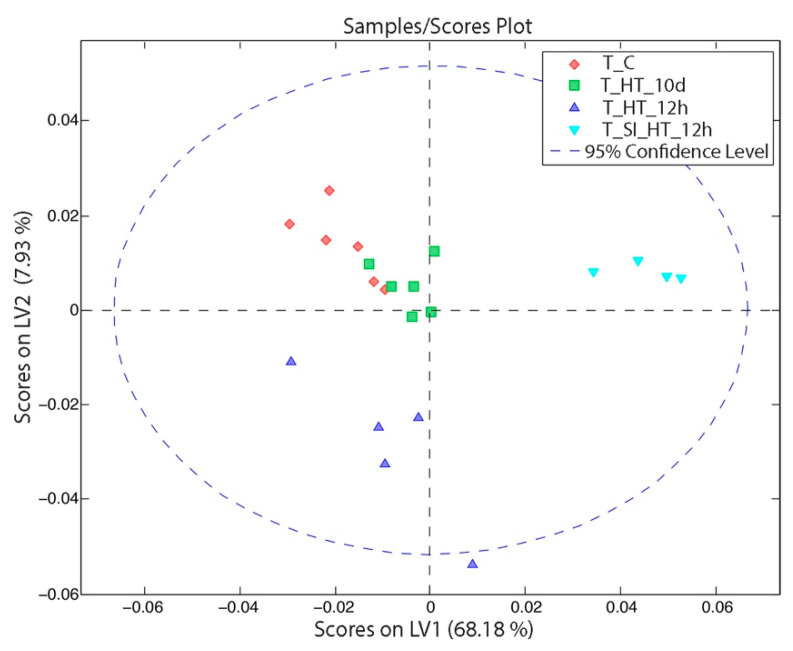
OPLS-DA scores from a dataset that combines tumor tissues from rats that suffered RCLM (TC, THT10d, THT12h and TSIHT12h) under different procedures. Key information to understand the abbreviations: control (C), tumor (T), hyperthermia therapy procedure (HT), sham surgery (SI) and period of time elapsed since the procedure (12 h or 10 d).

In the same manner that we studied the hepatic tissue samples, we built a new OPLS-DA model to observe the clustering of tumor samples after the different treatments. In Figure 7, the three main groups can be observed in the score plot: the tumor tissue 12 h after HT at the low part of the plot, the tumor tissue 12 h after the sham operation and HT at the upper right-hand, and the last group where tumor control tissue appears together with the tumor tissues 10 d after HT.

**Figure 8:**
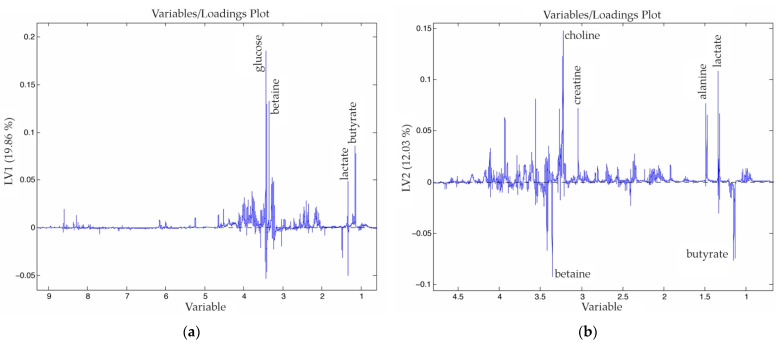
Loadings of the first (**a**) and second (**b**) PC of the OPLS-DA analysis obtained from a dataset that combines tumor tissues from rats that suffer CRCLM under different procedures.

Regarding to the loadings of the model (Figure 8), in the first PC, four signals were increased (glucose, betaine, lactate and butyrate) that characterize the positive right-hand side of the score plot (Figure 8a). In the second PC (Figure 8b), which helps us to distinguish the tumor tissues that underwent HT, we find that the betaine, choline, creatine, alanine, lactate and butyrate signals increased. As we have done in the previous cases, we focused on the VIPs to study the profiles of each class.

In Table A4 and Figure S4, one signal that stands out is the α-glucose in the case of tumors that underwent both treatments. The tumor tissue showed a completely different behavior if we compare it with the healthy hepatic tissue. Among the most important metabolic differences that can be observed in Table A4 are the reduction of lactate and an important increment of glycine in both the T_HT_12h and T_SI_HT_12h groups. Additionally, the tumor tissue from rats with HT and SI was especially characterized by an increment of valine, pyruvate, glutathione (oxidized), anserine, glycine and α-glucose.

The tumor tissue that underwent HT treatment still showed important modifications in its metabolic profile 10 d after treatment. That is, most of the metabolite’s levels decreased, while a few other metabolites, such as D-hydroxybutyrate, lactate, creatine and betaine, were enhanced.

## 4. Discussion

Previously, using the same electromagnetic applicator, under the same experimental conditions as in the present piece of work, and with Fe_3_O_4_ magnetic nanoparticles, we had already demonstrated that MHT achieves about 20% tumor destruction, while only 4% tumor destruction was observed in those animals that did not receive NPs [11]. In this piece of work, our aim was to assess whether the exposure to EMF, such as that required for HT therapy, could affect the normal functions of the liver.

First of all, we observed that tumor induction and development itself generated increases in the levels of certain metabolites detected in the liver parenchyma, such as betaine, taurine, TMAO or phosphatidylcholine. The increase of these metabolites is in accordance with results previously published, which associated the disruption of the serum levels of these molecules with patients who suffered from CRC [45,46]. These metabolites are products of the choline phospholipid metabolism, which, as seen in Table A2 and Figure 4, appears to have been altered in the healthy liver tissue of rats with CRCLM.

For example, an increased betaine level, which is derived from choline oxidation (methyl donor group), indicates an increase in methylation reactions, which is important in cell replication processes, detoxification reactions in the liver, or protection against liver damage. We also found an increase in both glutathione and ascorbic acid (Table A2), both with antioxidant properties, which leads us to believe that the liver tissue suffered from oxidative stress processes [47,48]. The liver needs to increase its metabolism in order to survive and maintain integrity, and therefore increased glycogen consumption was observed as an increase in glucose demand. On the other hand, in the tumor tissue, some amino acids, such as aspartate, leucine/isoleucine, valine, alanine, glycine or lysine, increased compared to healthy liver tissue of animals with non-carrier tumors. This is related to a high rate of tumor cell proliferation [49,50].

In general, the metabolic pathways involved in energy generation are also altered in cancer-related processes [51], including glycolysis [52,53], mitochondrial biogenesis [54], glutaminolysis [53], etc., which is consistent with the detection of high levels of metabolites involved in these pathways, including glucose, glutamine, pyruvate, citrate, N-acetylated compounds, etc. Glycolysis was historically believed to be a pathway used by tumor cells to obtain energy [50,51], a phenomenon commonly referred as the Warburg effect. However, there is increasing evidence that glycolysis is an adaptation of tumor cells to hypoxic conditions and that the glycolysis confers a significant growth advantage by producing the metabolites necessary for cancer cell growth [55].

When metabolomic analyses are performed in both hepatic tissue from tumor- and non-tumor-bearing animals of all of the experimental groups, we observed three different clusters based on the profile of the rats’ liver metabolism (Figure 5). One of them contained the samples obtained from control animals and all of the samples obtained 10 d after the procedures, while the other two clusters included samples that were obtained 12 h after the last procedure. One of these last clusters contained the samples from animals in which sham operations (RT_SI_HT_12h) were performed to mimic MNP-RGD infusion (cluster located on the upper right corner). The samples from animals exposed to EMF were grouped in the third cluster (located on the left side); it is noteworthy that the samples from animals receiving both sham operations and EMF exposure were also located in this last cluster. Thus, it seems that exposure to EMF had a different impact on the alteration of the metabolomic profile of liver tissue than the sham operation. This clear differentiation suggests that the metabolic answer for each type of stress is different. This class clustering suggests that the metabolome profile offers a good assessment of the stressing effects. Unfortunately, due to the lack of similar studies, we are not able to fully substantiate our findings.

In these experiments, when applying the described experimental condition, HT in the electromagnetic field did not induce chronic or non-reversible changes in the animals’ metabolism. Ten days after therapeutic exposure to EMF, the animals showed a metabolic profile that was quite similar to basal ones (Figure 5).

In the rats that underwent HT therapy, the pyruvate (P) formed lactate (L) instead of acetyl-CoA, which suggests a malfunctioning of the TCA cycle while the glycolysis works fine. One clear evidence of this is the increment of lactate proportion (which was the double). Therefore, we can conclude that there is reduced mitochondrial activity under HT. In the case of the sham operation (SI_12h) we observed an increment of lactate as a consequence of the injury caused during the surgery and the sham surgery that could damage the endothelium. However, the pyruvate–lactate ratio was still normal.

In the samples obtained 12 h after EMF exposure, a high L:P rate was shown (six times higher than healthy hepatic tissue), which indicates an acute hepatic failure [56,57,58]. However, when looking at samples obtained 10 d after the last procedure, we found that the L:P rate was normalized, which indicates an evident recovery of liver function.

The L:P ratio reflects the balanced or unbalanced state between the product and the substrate of the reaction catalyzed by LDH; it also reflects, indirectly, the NADH:NAD+ ratio, which allows us to know that the redox state of the liver cells’ cytoplasm is altered, with an excess of reduced equivalents (NADH >> NAD^+^) [59].

The metabolic response patterns or clusters observed, depending on the different surgical procedures, were reinforced by the serum enzyme data obtained from the blood samples (Figure 1 and Table S1). All of the serum enzymes tested, apart from creatinine, showed different variations depending on the surgical procedure, but these changes were only found in the experimental groups analyzed 12 h after the last procedure.

The creatinine values remained without variations between the experimental groups, and within the reference values (0.4–1.5 mg/dL), which indicates that the exposure to HT and the surgical procedures did not cause lysis of the muscular tissue or alterations in its metabolism [60,61].

The LDH serum levels measured in the first 12 h showed evidence of an imbalance in the L:P, a fact that we had already observed with the metabolomic analyses. This enzyme catalyzes the conversion of lactate to pyruvate, and vice versa, through the oxidation of NADH to NAD^+^, thus maintaining an adequate L:P ratio and an optimal redox state of the cytoplasm. It is expected that in the case of a decompensation of the L:P ratio, an excess of LDH activity would occur and it would be detected in the serum analysis [58,59].

Levels of ALP, an ubiquitous enzyme that can indicate both liver and bone diseases or other disorders [62], were within the range in accordance with published data (82–164 UI/L) [63]. In spite of this, we noticed that the ALP values measured after 10 d were significantly lower than the basal values of the control animals. However, none of the procedures performed could explain this decrease, since in the literature consulted this decrease is attributable to treatment with estrogens in women with osteoporosis, children with achondroplasty, post-cardiac surgery, hypothyroidism, chronic myeloid leukemia, Wilson’s disease, treatment with contraceptives, etc. [64,65].

Finally, when considering enzymes that could indicate any liver disorder, both AST and ALT displayed the same trend, although in different magnitudes. Analyzed 12 h after the treatment, the increase in serum AST values was three times higher than ALT. This disparity in the magnitude of the detected change of both transaminases is due to the ubiquitous presence of the AST, which can also be increased when there is some damage in the myocardium, skeletal muscle, kidney or brain [61]. However, the most important aspect of these results is that, as it happened with the other enzymes, molecules or metabolites, their levels after 10 d returned to normal values.

In the same way that we have studied the last two cases, we built a new OPLS-DA model (Figure 7 and Figure 8) to observe the clustering of tumor samples after the different treatments. The tumor tissue showed a completely different behavior when compared to the hepatocellular tissue. After 10 d, the tumor cells that underwent hyperthermia treatment still showed important differences in the metabolic profile. Most of the metabolite’s levels decreased while a few metabolites were enhanced, such as D-hydroxybutyrate, lactate, creatine and betaine. Additionally, the tumor tissues that belonged to the rats after HT and sham-surgery were especially characterized by the highest values of glutathione (oxidized), which can suggest that this group suffered the highest stress.

## 5. Conclusions

In summary, exposure to electromagnetic fields of the same magnitude as those generated during the application of magnetic hyperthermia therapy produces, during the early postoperative period, alterations, both at the local level of liver tissue and in the body’s metabolism as a whole. However, after a period of recovery, the basal metabolism of animals exposed to HT begins to recover.

## Data Availability

The data that support the findings of this study are available from the corresponding author, (B.H.d.l.P and M.I.), upon reasonable request.

## Supplementary Materials

The following are available online at https://www.mdpi.com/article/10.3390/nano11051318/s1, Figure S1: The signal increment of glucose, pyruvate, lactate, citrate, fumarate and D-hydroxybutyrate obtained from the VIP showed graphically and referred to the healthy tissue (C). Key information to understand the abbreviations: control (C), hepatic tissue (H), rat with CRCLM (RT), hyperthermia therapy procedure (HT), sham surgery (SI) and period of time elapsed since the procedure (12 h or 10 days)., Figure S2: The signal increment of glutathione, choline, betaine and asparagine obtained from the VIP showed graphically and referred to the healthy tissue (C). Key information to understand the abbreviations: control (C), hepatic tissue (H), rat with CRCLM (RT), hyperthermia therapy procedure (HT), sham surgery (SI) and period of time elapsed since the procedure (12 h or 10 days), Figure S3: The signal increment of the most meaningful metabolites obtained from the VIP showed graphically and referred to the healthy tissue (C). Key information to understand the abbreviations: control (C), hepatic tissue (H), rat with CRCLM (RT), hyperthermia therapy procedure (HT), sham surgery (SI) and period of time elapsed since the procedure (12 h or 10 days), Figure S4: The signal increment of a-glucose, lactate, D-hydroxybutyrate, choline, betaine, glycine, taurine & trimethylamine-N-oxide and glutathione (oxidized) obtained from the VIP showed graphically and referred to the tumor control tissue (TC). Key information to understand the abbreviations: control (C), tumor (T), hyperthermia therapy procedure (HT), sham surgery (SI) and period of time elapsed since the procedure (12 h or 10 days).

## Appendix A

**Table A1:** Biochemical serum levels. Mean and SD values of the blood plasma levels of the different enzymes analysed: alanine transaminase (ALT), aspartate transaminase (AST), alkaline phosphatase (AP), amylase, creatine kinase (CK), lactate dehydrogenase (LDH), and creatinine (Cr). The units of measurement for each enzyme are shown in brackets: international units per litre (IU/l), and milligrams per decilitre (mg/dl). Key information to understand the abbreviations: control (C), rat with CRCLM (RT), hyperthermia therapy procedure (HT), sham surgery (SI) and period of time elapsed since the procedure (12 h or 10 days).

Experimental Group	ALT (IU/l)	AST (IU/l)	AP (IU/l)	Amylase (IU/l)	CK (IU/l)	LDH (IU/l)	Creatinine (mg/dl)
C	42 ± 4.6	57 ± 3.61	158 ± 16.5	2361 ± 152	98.3 ± 14.2	63.7 ± 27.6	0.413 ± 0.035
RT	46 ± 4	94.3 ± 21.9	119 ± 8.7	2083 ± 140	115 ± 8.5	72.7 ± 9.3	0.397 ± 0.038
SI_12h	128 ± 38	202 ± 45.8	128 ± 8.7	1349 ± 231	180 ± 66.1	208 ± 118	0.384 ± 0.057
HT_12h	106 ± 34	370 ± 73.9	194 ± 30.4	1410 ± 114	191 ± 51.2	172 ± 42.7	0.454 ± 0.057
RT_HT_12h	124 ± 77	388 ± 119	156 ± 26	1503 ± 121	129 ± 96.4	179 ± 58.9	0.462 ± 0,091
RT_SI_HT_12h	129 ± 73	331 ± 121	124 ± 24.3	1928 ± 180	265 ± 82.7	170 ± 1.53	0.462 ± 0.135
HT_10d	41 ± 7	61 ± 11.4	111 ± 21.9	1554 ± 181	98.4 ± 11.4	61.7 ± 26.1	0.385 ± 0.054
RT_HT_10d	38 ± 5.2	89.7 ± 20	115 ± 14	2303 ± 227	100 ± 18.8	66.2 ± 11.4	0.328 ± 0.053
RT_SI_10d	42 ± 6.6	58.7 ± 14.6	95 ± 7.6	1692 ± 184	93.8 ± 17.2	73.8 ± 30.4	0.408 ± 0.043

**Table A2:** The signal increment of the most meaningful metabolites obtained from the VIP showed in a heatmap referred to the healthy tissue (C). Key information to understand the abbreviations: control (C), hepatic tissue (H), rat with CRCLM (RT) and tumour (T).

Metabolite	ppm	C	H_RT_C	T
leucine/isoleucine	0.966	1.00	0.57	1.26
valine	0.984	1.00	0.12	1.53
D-hydroxybutyrate	1.187	1.00	2.23	0.30
lactate	1.325	1.00	0.85	1.29
alanine	1.492	1.00	0.99	1.09
lysine	1.719	1.00	0.46	1.34
acetate, acetoacetate	1.921	1.00	2.36	0.22
N-acetyl functions of glycoprotein	2.053	1.00	0.31	1.43
glutamine	2.152	1.00	0.73	1.15
glutamate	2.359	1.00	0.37	1.40
pyruvate	2.408	1.00	0.24	1.39
gluthathione (oxidized)	2.559	1.00	1.46	0.77
aspartate	2.805	1.00	0.46	1.34
creatine	3.040	1.00	0.43	1.52
choline	3.225	1.00	0.17	1.39
GPC (sn-glycerol-3-phosphocholine)	3.233	1.00	0.52	0.77
PC (phosphatidylcholine)	3.250	1.00	1.38	0.40
taurine, trimethylamine-N-oxide	3.271	1.00	2.04	0.13
betaine	3.359	1.00	2.51	1.34
𝜶-glucose	3.424	1.00	1.18	1.43
glycine	3.553	1.00	0.59	1.43
citrate	3.562	1.00	0.20	1.43
threonine	3.609	1.00	0.22	1.46
free glycerol	3.651	1.00	0.17	1.49
ascorbic acid	4.504	1.00	2.42	0.20

**Table A3:** The signal increment of the he signal increment of the most meaningful metabolites obtained from the VIP showed in a heatmap referred to the healthy tissue (C). Key information to understand the abbreviations: control (C), hepatic tissue (H), rat with CRCLM (RT), hyperthermia therapy procedure (HT), sham surgery (SI) and period of time elapsed since the procedure (12 h or 10 days).

Metabolite	ppm	C	H_RT_C	H_HT_12h	H_RT_HT_12h	H_RT_SI_HT_12h	H_SI_12h	H_HT_10d	H_RT_HT_10d
leucine/isoleucine	0.966	1.00	0.30	0.46	0.34	0.50	0.81	1.18	0.47
valine	0.984	1.00	0.19	0.23	0.17	0.28	0.72	1.20	0.38
D-hydroxybutyrate	1.187	1.00	2.24	11.39	6.54	10.84	5.39	0.95	2.62
lactate	1.325	1.00	0.81	3.24	1.94	3.12	1.86	1.23	0.98
alanine	1.492	1.00	1.60	1.39	1.36	1.36	1.15	0.92	1.26
lysine	1.719	1.00	0.20	0.21	0.17	0.27	0.71	1.20	0.39
acetate, acetoacetate	1.921	1.00	1.47	0.22	0.87	0.24	0.59	0.84	1.27
glutamate	2.133	1.00	0.41	1.22	0.68	1.23	1.14	1.21	0.52
pyruvate	2.408	1.00	0.26	0.49	0.33	0.53	0.83	1.19	0.44
glutamine	2.480	1.00	0.21	1.43	0.74	1.42	1.23	1.23	0.48
gluthathione (oxidized)	2.565	1.00	0.91	3.91	2.06	3.77	2.24	1.17	1.04
asparagine	2.960	1.00	0.46	2.86	1.53	2.78	1.84	1.21	0.75
creatine	3.040	1.00	0.43	0.87	0.60	0.89	0.98	1.16	0.59
choline	3.225	1.00	2.75	9.24	5.83	8.78	4.43	0.84	2.82
GPC (sn-glycerol-3-3-phosphocholine)	3.233	1.00	0.37	0.55	0.42	0.59	0.85	1.17	0.52
PC (phosphatidylcholine)	3.250	1.00	1.14	3.53	1.85	1.63	2.02	3.41	2.10
betaine	3.359	1.00	2.25	4.65	3.41	4.44	2.49	0.76	2.12
𝜶-glucose	3.424	1.00	3.07	4.07	2.84	3.87	1.93	0.43	2.45
glycine	3.553	1.00	1.35	6.01	3.26	5.75	3.04	1.07	1.22
citrate	3.562	1.00	0.53	0.25	0.31	0.30	0.73	1.22	0.53
threonine	3.609	1.00	1.01	0.57	0.81	0.59	0.81	1.00	0.98
free glycerol	3.651	1.00	1.77	13.50	7.19	12.83	6.07	1.12	2.29
glucose-1-phosphate-(glycogen)	4.504	1.00	0.00	0.22	0.07	0.28	0.73	1.25	0.26
ascorbic acid	4.515	1.00	0.26	0.92	0.48	0.94	1.02	1.23	0.41
glycogen	5.416	1.00	0.01	0.15	0.03	0.22	0.70	1.25	0.25

**Table A4:** The signal increment of the most meaningful metabolites obtained from the VIP showed in a heatmap referred to the healthy tissue (C). Key information to understand the abbreviations: control (C), hepatic tissue (H), rat with CRCLM (RT) and tumour (T).

Metabolite	ppm	T_C	T_HT_10d	T_HT_12h	T_SI_HT_12h
leucine/isoleucine	0.966	1.00	0.28	2.76	0.21
valine	0.984	1.00	0.28	0.15	2.57
D-hydroxybutyrate	1.187	1.00	2.39	0.43	0.17
lactate	1.325	1.00	2.04	0.75	0.32
alanine	1.492	1.00	0.43	3.45	0.31
lysine	1.719	1.00	0.52	2.14	0.23
acetate, acetoacetate	1.921	1.00	0.46	2.41	0.51
N-acetyl functions of glycoprotein	2.053	1.00	0.20	2.14	0.85
pyruvate	2.408	1.00	0.18	1.22	2.51
gluthathione (oxidized)	2.559	1.00	0.27	1.11	1.83
aspartate	2.692	1.00	0.22	2.24	0.77
dimethylglycine	2.941	1.00	0.61	1.38	1.15
anserine	2.962	1.00	0.67	0.89	1.43
creatine	3.042	1.00	1.58	1.66	0.24
choline	3.225	1.00	1.04	1.94	0.14
GPC (sn-glycerol-3-phosphocholine)	3.233	1.00	0.12	2.40	0.62
taurine, trimethylamine-N-oxide	3.271	1.00	0.23	2.55	0.99
glycine	3.555	1.00	0.65	1.71	1.61
betaine	3.359	1.00	1.90	0.16	0.19
𝜶-glucose	3.424	1.00	0.32	0.48	8.09
citrate	3.562	1.00	0.92	1.95	0.31
