# Supplementary material for: Biochemical and Metabolomic Changes after Electromagnetic Hyperthermia Exposure to Treat Colorectal Cancer Liver Implants in Rats
Source: Nanomaterials (Basel). 2021 May 17;11(5):1318. doi: 10.3390/nano11051318 (PMC8156717; doi:10.3390/nano11051318)
Supplement: Supplementary file 1 [file nanomaterials-11-01318-s001.zip › nanomaterials-1203592-supplementary.pdf]

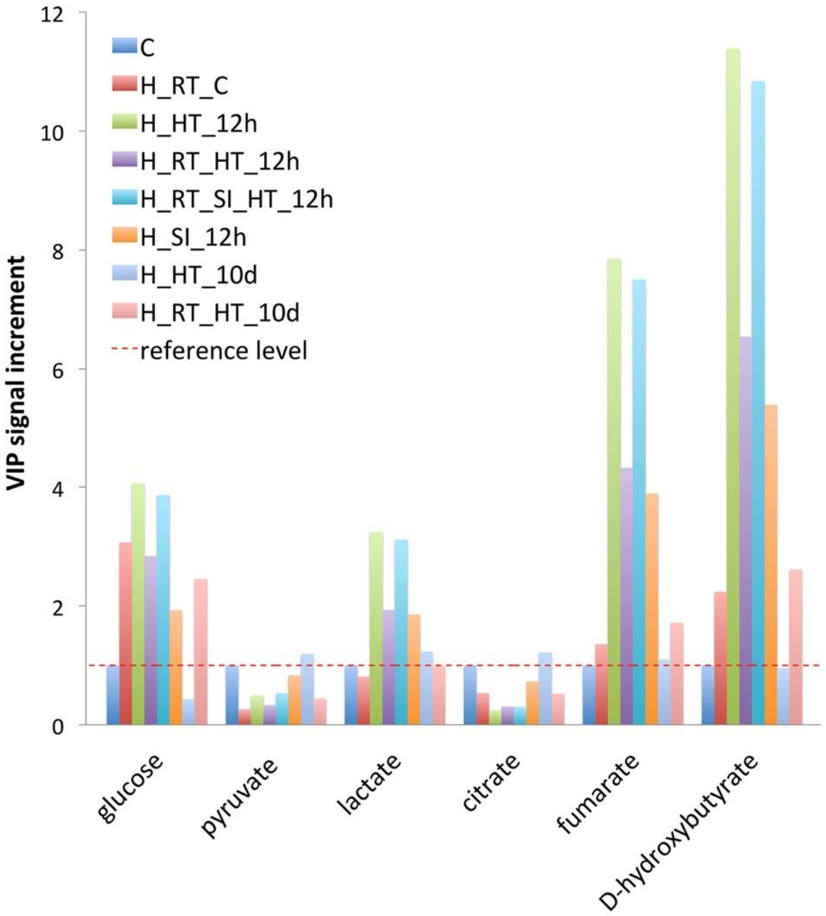

Figure S1. The signal increment of glucose, pyruvate, lactate, citrate, fumarate and D-hydroxybutyrate obtained from the VIP showed graphically and referred to the healthy tissue (C). Key information to understand the abbreviations: control (C), hepatic tissue (H), rat with CRCLM (RT), hyperthermia therapy procedure (HT), sham surgery (SI) and period of time elapsed since the procedure (12 hours or 10 days).

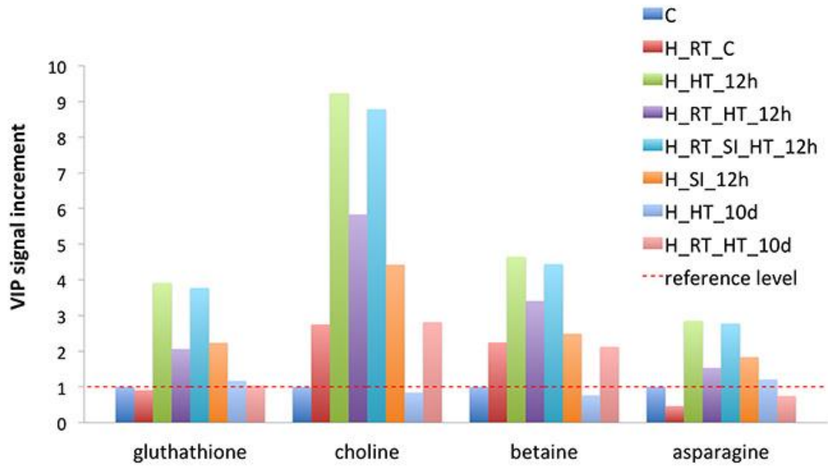

Figure S2. The signal increment of glutathione, choline, betaine and asparagine obtained from the VIP showed graphically and referred to the healthy tissue (C). Key information to understand the abbreviations: control (C), hepatic tissue (H), rat with CRCLM (RT), hyperthermia therapy procedure (HT), sham surgery (SI) and period of time elapsed since the procedure (12 hours or 10 days).

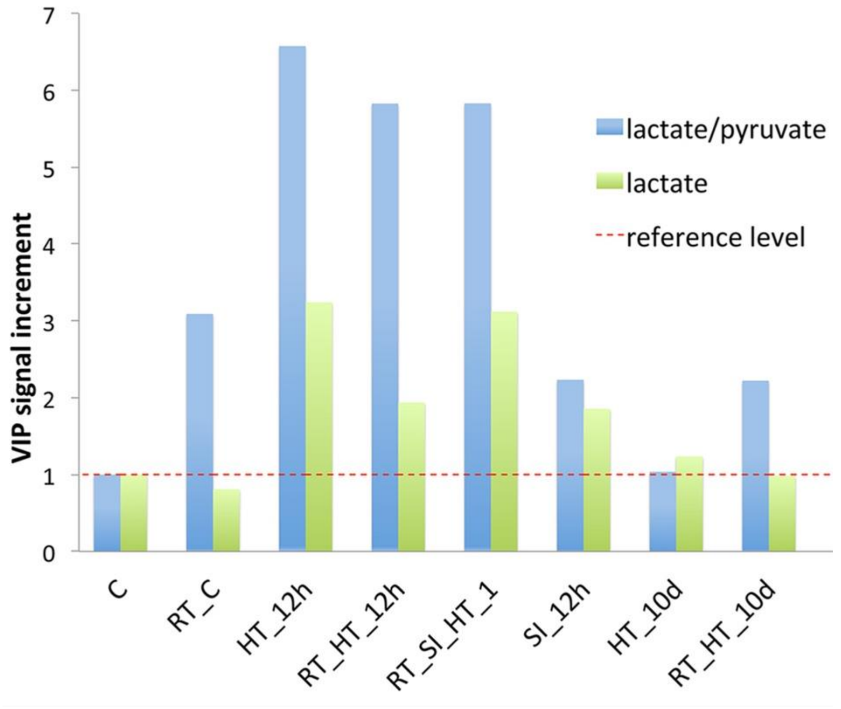

Figure S3. The signal increment of the most meaningful metabolites obtained from the VIP showed graphically and referred to the healthy tissue (C). Key information to understand the abbreviations: control (C), hepatic tissue (H), rat with CRCLM (RT), hyperthermia therapy procedure (HT), sham surgery (SI) and period of time elapsed since the procedure (12 hours or 10 days).

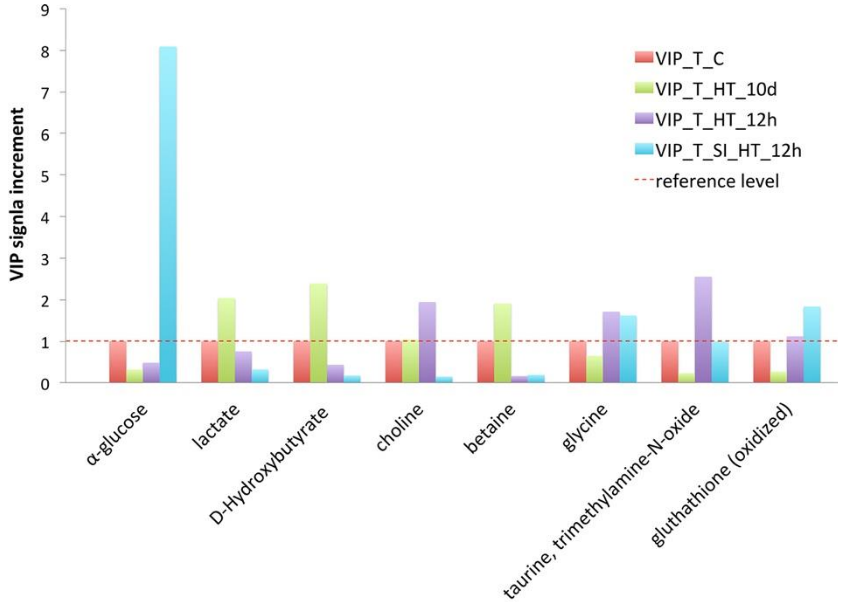

Figure S4. The signal increment of a-glucose, lactate, D-hydroxybutyrate, choline, betaine, glycine, taurine & trimethylamine-N-oxide and glutathione (oxidized) obtained from the VIP showed graphically and referred to the tumour control tissue (TC). Key information to understand the abbreviations: control (C), tumour (T), hyperthermia therapy procedure (HT), sham surgery (SI) and period of time elapsed since the procedure (12 hours or 10 days).
